# Solid pseudopapillary neoplasm (SPN) of the pancreas: current understanding on its malignant potential and management

**DOI:** 10.1007/s12672-024-00905-5

**Published:** 2024-03-18

**Authors:** Xiaoyue Lu, Hao Chen, Taiping Zhang

**Affiliations:** 1 Peking Union Medical College, Beijing, China; 2 Department of General Surgery, Chinese Academy of Medical Sciences, Peking Union Medical College Hospital, Beijing, China

**Keywords:** Solid pseudopapillary neoplasm, Malignant potential, Identification and assessment, Management, Prognosis

## Abstract

Solid pseudopapillary neoplasms (SPN) of the pancreas are presently recognized as low-grade malignant tumors that are frequently observed in young females. This tumor has a low incidence and is associated with an excellent prognosis following surgical resection. Typical SPNs primarily affect the pancreas and tend to have moderate or asymptomatic manifestations. Based on retrospective research, it is anticipated that patients with SPN can achieve disease-free survival, even in cases when metastasis is detected during inspection. However, the incidence of malignant SPN has been consistently underestimated, as evidenced by recent research findings. Malignancy of SPN primarily encompasses invasion and infiltration, metastasis, and recurrence after R0 resection. Imaging technologies such as Ultrasound, Computed Tomography, Magnetic Resonance Imaging, and Position Emission Tomography are capable of preliminarily identifying malignant SPN, which is primarily based on its invasive clinical features. Research on risk factors of malignant SPN revealed that larger tumor size, Ki-67 index, and several other parameters had significant correlations with invasive tumor behavior. Pathologic features of malignant SPNs overlay other pancreatic tumors, nevertheless they can provide valuable assistance in the process of diagnosis. Several confirmed specific pathologic biomarkers are related to its cellular origin, characteristic gene mutation, and cell proliferation. Considering the invasiveness of malignant SPN, it is imperative to enhance the comprehensiveness of its therapy. Tumor resection remains a suggested course of action in line with typical SPN, and additional lymph node dissection is seen as reasonable. Compared to benign SPNs, malignant SPNs have worse prognosis, underscoring the necessity of early identification and treatment in comprehensive medical centers to get improved clinical outcomes.

## Introduction

Solid pseudopapillary neoplasm (SPN) of the pancreas is a rare solid or cystic tumor first described by Frantz in 1959 [1]. The tumor, previously known by various names such as Frantz’s neoplasm, Hamoudi’s neoplasm, papillary cystic neoplasm, solid and papillary epithelial neoplasm, and solid and cystic papillary epithelial neoplasm, was officially classified as solid pseudopapillary tumor by the World Health Organization in 1996, and further categorized as solid pseudopapillary neoplasm in 2010 [2]. The incidence rate of SPN is low, with a reported proportion of 2–3% in all pancreatic neoplasms [3]. Nevertheless, there has been a notable surge in documented cases of SPN, with a sevenfold increase observed since the year 2000. This upward trend can potentially be attributed to improvement in our understanding of related knowledge and medical diagnostic techniques [4]. A single-center clinical review from China in 2018 reported that SPN accounts for 2.15% of total pancreatic surgeries [5]. SPN displays an incidence preference for young females (up to 2–14 folds more than males) implicating the relationship between its development and specific hormones or receptors [6–8]. A case series study from Fudan University showed that the sex ratio of SPN in China is 58:13 (female: male) [9]. Besides the incidence rate, the two genders display a noticeable disparity in average morbidity age. According to Wu’s research utilizing the SEER database, 49.2% of male SPN patients were diagnosed older than 65 years old, while 80.1% of female patients were diagnosed before 65 years old [7]. Another research conducted by Hruban reported that the mean age of female SPN patients is about 28, and the proportion of SPN in all pancreatic neoplasms in females under 40 is around 30% [10]. The statistical data currently available indicates that there is a notable distinction in the sex ratio between adolescent patients and adults. Mylonas observed that 77.3% of adolescent SPN patients are female, which indicates a larger proportion of females in comparison to adult patients [11]. Racial disparities are rarely mentioned in literature analyzing the incidence rate of SPN, but a national study of 369 patients at Duke University Medical Center found that individuals of white ethnicity exhibit greater susceptibility to SPN [12].

SPN mainly occurs in the pancreatic tail, occasionally observed in the head or other parts of the pancreas in adults, which is the most common age group. In contrast to older patients, young adolescents tend to experience SPN mostly in the head of the pancreas [11]. SPN is asymptomatic under most circumstances. Therefore, it is usually discovered in routine medical examinations. A limited cohort of individuals exhibits some unspecific symptoms such as nausea, vomiting, abdominal discomfort, pain, asthenia, fever, jaundice, and weight loss [13]. Patients with large neoplasms may experience acute abdomen resulting from traumatic intratumoral hemorrhage [10].

## Identification and assessment of SPN malignancy

### Definition and incidence of SPN malignancy

SPN has been perceived as a low-grade malignant tumor with a good prognosis proved by a reported 10-year disease-free survival rate of 94% and a local recurrence rate of less than 10% [14]. After R0 surgical resection, a significant number of patients, even those with symptoms of metastasis, can attain prolonged periods of disease-free survival [15]. The underestimation of the malignant potential of solitary pulmonary nodules (SPN) has persisted due to the perceived low probabilities of metastasis and vascular invasion. However, accumulated studies gradually suggested that SPN was more than a low-aggressive tumor. For instance, Tang’s research indicated that metastasis actually occurs in 10–15% of patients based on pathological results after surgical resection [16]. For patients with certain risk characteristics which involves around 45% of all SPN cases, the 10-year RFS decreased to 75.3% [17]. Adolescents are frequently presented with intricate cases characterized by the presence of sizable tumors, local invasion, and metastasis. Therefore, a more comprehensive comprehension is required to improve our concept of SPN and apply personalized treatments.

**Fig. 1:**
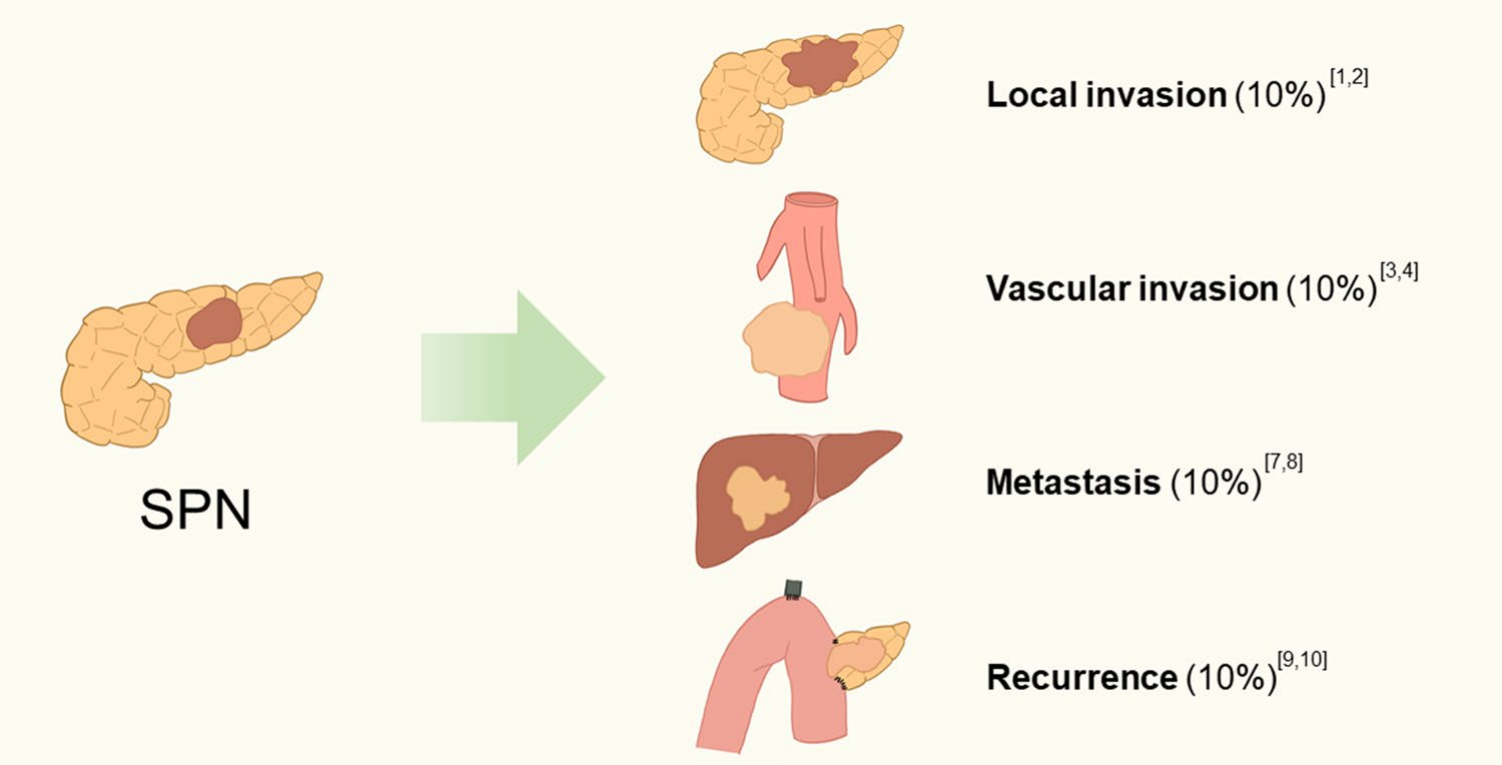
Malignancy performance and incidence rate

A clear definition of malignancy for SPN is deficient. According to previously published clinical studies and literature reviews, a set of criteria was proposed. Most researchers commonly employ comparable criteria to evaluate other pancreatic neoplasms, which include infiltration to adjacent tissues, metastasis, and recurrence after R0 resection. WHO suggested in *2000 tumor classification* that perineural invasion, angioinvasion, or deep invasion into surrounding tissues could be defined as malignant development [18]. However, the evidence of malignant SPN varies in different clinical studies. Song found that SPN is always defined as malignant when symptoms such as vascular infiltration, pancreatic parenchymal invasion concurrent with peripancreatic fat tissue infiltration, adjacent organ invasion, and perineural invasion are presented [19]. In a study based on the Memorial Sloan-Kattering Cancer Center Department of Surgery’s prospective pancreatic database in 15 years, Martin observed that three out of 24 SPN patients exhibited vascular invasion, one patient developed liver metastases, and one presented with local peritoneal metastasis [20]. The analysis of Hao demonstrated that systematic metastasis, local recurrence, deep invasion, and unresectability were commonly detected in aggressive SPNs [21]. Reindl's case report showed atypical histologic findings as evidence of malignancy, including extensive necrosis, prominent nuclear atypia, elevated mitotic count, and high Ki-67 index [22]. Capsular infiltration was also classified as malignancy in another review [23]. In conclusion, tumor behaviors similar to other malignancies and poor differentiation indicate strong malignant tendencies (see Fig. 1).

### Identifying SPN malignancy with imaging approaches

Identifying malignant SPNs based on imaging results exhibits a somewhat lower but still guaranteed accuracy compared to some other neoplasms due to their unspecific morphological characteristics. Ultrasound, CT, MRI, and PET-CT are often employed modalities for the diagnosis and assessment of SPN. SPN is usually observed as a hypoechoic mass with a homogeneous or heterogeneous structure under ultrasound. The primary advantages of US are accessibility, convenience, and lower costs without radiation. When a malignant SPN encircles the main artery around the pancreas, transabdominal ultrasound will be the most convenient way for initial resectability assessment. Recent advancements in ultrasound technology have demonstrated enhanced precision than conventional US in the identification of SPNs. For example, contrast-enhanced ultrasound (CEUS) can help reveal tumor margin perfusion in the arterial phase and analyze malignancy of lesions based on peak enhancement patterns [24].

CT is the mainstream radiological examination for SPN. The variation of CT attenuation value between solid, cystic, and hemorrhagic areas is obvious. The characteristic appearance of SPN under CT is encapsulated mass with irregular solid and cystic components, in which calcifications and solid portions can be detected [25]. Multiphasic CT distinguishes solid structure from pancreas parenchyma according to weak early arterial enhancement and strong enhancement in the portal-venous phase [20]. Some literature has indicated that CT had unique advantages compared to the US when focusing on malignant SPNs [26]. CT has a higher resolution which is less dependent on the technicians or radiologists, therefore enables a comprehensive evaluation of the integrity of the tumor capsule, which may indicate potential malignant behavior. Gopinathan reported in a review of 10 patients from a single institution that 2 cases with spleen invasion are visible of breach of fibrous pseudocapsule [27]. Direct recognition of tumor invasion to adjacent blood vessels and tissue is also feasible with CT. The primary application of advanced CT technologies such as 3D-reconstruction CT in clinical aspects is to assist surgical procedures and to focus on vascular distribution near the tumor. Huang studied 85 SPN patients and discovered that 3D models were more accurate than 2D models in identifying malignant SPNs. They constructed six logistic regression models using radiomics features extracted from radiologic images, and the results pointed out that arterial radiomics model constructed by 3D-ROI features may predict metastasis of malignant SPN [28]. A nomogram model for MSCT established by Li incorporated tumor growth pattern, annular enhancement, capsule, and CT value of lesion in the arterial phase. The model achieved promising accuracy in the preoperative prediction of invasion of SPN [26]. No research on the usage of dual-energy CT in diagnosing SPN has been conducted, but this emerging technique allows us to yield images of higher quality and mitigate the presence of artifacts.

Compared with CT, the main advantage of MRI is its better resolution on soft tissue. Consequently, there will be a significant advancement in the discrimination on the relationship between tumor and surrounding tissue. Extensive hemorrhagic lesions account for heterogeneous signal intensity on T1 and T2 weight images [29]. Underlying hemorrhage may cover soft tissue enhancement in SPN, and MR subtraction imaging should be carried out to solve this problem [30]. Invasion of malignant SPN is shown as a tumorous mass in normal tissue range such as liver and peritoneum, therefore CT and MRI are preferred methods of differential diagnosis on the malignancy of SPN. A retrospective study including 132 cases of definitely radiologically diagnosed SPN indicated that the overall diagnosis rate was 78.5% for CT and 77.8% for MRI [31]. MRCP result is able to show communication between the lesion and the tumor but has limitations when focusing on recognizing malignant SPNs [32].

SPN has strong and consistent avidity for ^18^F-FDG, facilitating PET-CT for recognizing them (the specific value of SUVmax for ^18^F-FDG varied largely, depending on reasons such as the manufacturer of the machine) [33]. The metastatic region of malignant SPN shall also be detected by PET-CT based on this distinction. The mechanism of increased glucose uptake in SPN remains unclear. Generally, the high glucose uptake level in cancer cells can be attributed to their elevated requirement of energy, which definitely applies to SPN. In addition, Sato found that SPN has enhanced GLUT-1 expression, leading to alterations in tumor cell density and mitochondria [34]. Some new mechanisms for this phenomenon have been discovered in recent years, such as the theory of impaired degradation of β-catenin, which stimulates glucose transport mediated by SGLT1 and SGK1 [35–37]. No consensus has been introduced when it comes to the reason for upgraded glucose intake in malignant SPNs. However, in Kim’s review of 10 SPN patients from Yousei University Severance Hospital, indexes including mean SUV, metabolic tumor volume (MTV), total lesion glycolysis (TLG), and tumor-to-background ratio (TBR) are measured. Max SUV, mean SUV, MTV, and TLG values were found to correlate with the T stage of SPN [38]. Kang studied the medical records of 37 SPN patients and concluded that type III PET-CT configuration (multiple and geographic FDG uptake of the tumor) is an independent factor associated with invasive SPNs [33]. A possible explanation is that malignant SPNs differ from benign neoplasms enormously in metabolic pattern, which is possible to emerge in PET-CT. Besides glucose, 68 Ga-FAPI can indicate SPN as hyperabsorption lesions, while its use in defining malignancy remains unstudied [39]. Although not widely applied in clinical practice, the specific application of PET-CT in malignancy diagnosis is an emerging research orientation. Nevertheless, nonnegligible drawbacks including exorbitant price and low penetration rate may restrict its application. Similar to PET-CT, PET-MR combines the advantages of PET and MRI, displaying an elevated definition for SPNs. Despite being costly to the majority of patients, PET-MR can be employed to confirm identification before invasive examination [40] or to make a differential diagnosis [41].

### Risk factors for SPN malignancy

The risk factor of SPN malignancy is a controversial topic in the current research field. Consensual hazardous factors mainly focus on the cytological characteristics of the neoplasm, such as mitotic activity and cell pleomorphism. In the retrospective research of 63 SPN patients from the First Affiliated Hospital of Bengbu Medical College led by Chen, risk factors of recurrence and metastasis are large tumor size (diameter > 8 cm), a Ki-67 index over 5%, and lymph node metastasis [42]. Yang conducted a retrospective cohort study in two tertiary academic centers, in which analysis of 193 SPN patients provided additional evidence supporting the assistant effects of tumor diameter and Ki-67 index in predicting malignancy of SPN [43]. The connection between pathologic evaluation including tumor diameter plus Ki-67 index and malignant potential is also verified by Estrella in a systematic review of 64 cases of SPN conducted at a single institution [14]. Another multicenter research of Kang was conducted in 17 medical institutions, which chose pancreatic fat tissue invasion, capsular invasion, cellular atypia, perineural invasion, and lymph node metastasis to be the risk factors for SPN based on 351 patient data [44]. Lee made an analysis according to the latest worldwide standards for malignant SPN and found that risk factors for malignancy included large tumor size, lymphatic and vascular invasion, and synchronous metastasis [45]. Moreover, the pathology department team of Peking Union Medical College has recently developed a model to predict SPN malignant behavior, mainly containing tumor size, lymphovascular invasion status, and Ki-67 index [17]. Yang analyzed 25 formalin-fixed paraffin-embedded tissue samples and found invasive SPN group exhibited markedly increased infiltration of CD-68 positive TAMs and CD-163 positive M2 macrophages [46]. Combined, currently existing research results about risk factors for malignant SPN mainly focus on tumor size and parameters reflecting invasive cellular behavior. Nevertheless, there appear to be characteristics correlating with patient basic features. In a meta-analysis of 53 SPN patients from Song, younger age of adult patients is possibly linked with higher malignancy [19]. A univariate cox-regression analysis by Paredes showed that age ≥ 28 years, larger tumors ≥ 10 cm, invasion of adjacent organs, lymph node metastasis(pN +), and AJCC Stage III were factors able to predict recurrence of resected SPN [47].

## Pathological features of SPN: origin of malignancy

### Pathological examination in the diagnosis of SPN

Cytologic examination has become the prevailing method for evaluating patients suspected of SPN. A combination of solid and cystic structures could be observed under microscope. With small and round nuclei, mitosis is quite rare or even inexistent in pathological examination. Still, there exist certain scenarios in which distinguishing SPN from other types of pancreatic cancers becomes challenging. Higuchi reported in a case report about SPN mimicking adenoid cystic tumors because of representing microcystic configurations with mucus [48]. When special features are present, such as predominant acinar units, squamoid nests, prominent central nucleoli, granular eosinophilic cytoplasm enclosing DPAS-positive zymogen granules, and positive immunochemistry results for trypsin, chymotrypsin, BCL10, and lipase, it is possible to confuse SPN and pancreatoblastoma [49]. Other diseases that can mimic SPNs in clinical features include neuroendocrine neoplasms, acinar cell carcinoma [50], and autoimmune pancreatitis [51]. However, some distinct cellular characteristics become evident when SPN is defined as malignant. Zhang reported cellular pleomorphism and high nuclear grade as the microscopic features of malignant SPN [52]. After comparing two malignant SPN samples to 34 benign ones, Tang summarized that manifestations of malignancy include a diffuse growth pattern with extensive tumor necrosis, an unusually high mitotic rate, and the presence of an undifferentiated component such as sarcomatoid carcinoma elements [16].

The integration of pathologic biomarkers and morphological features is the gold standard for the diagnosis of SPN. However, upon individual analysis, pathologic markers of SPN overlap with other pancreatic neoplasms. Therefore, it is commonly used for auxiliary diagnosis. Limited by their cellular origins, each pancreatic neoplasm has its typical pathologic features. Although the cellular origin of SPN is still ambiguous, several biomarkers with high sensitivity have been discovered, which are correlated with its specific histological morphology. β-catenin, an important part of the Wnt pathway, is found to extensively increase in SPN specimens [53]. The designation of E-cadherin as a negative signal has been subject to new challenges in light of the latest research. Stefano discovered that applications of antibodies directly on the cytoplastic domain yield a positive E-cadherin detection result. However, SPN tumor cells show negative reactions when antibodies are targeted on extracellular segments [54]. Additional diagnostic biomarkers that could be considered include the Hedgehog pathway, Notch pathway, androgen receptor, and progesterone receptor [55].

As for malignant SPN, genes related to tumor migration were found to be specific in identifying these neoplasms. Some researchers from Italy analyzed a cohort of 27 surgically resected SPNs (22 normal and 5 metastatic), and found that genes relevant to inactivating epigenetic regulation are negatively expressed in metastatic SPNs, including BAP1 and KDM6A [56]. The high mitotic activity of SPN indicates the existence of undifferentiated carcinoma components. The rapid proliferation of these components contributes to the unfavorable prognosis of the tumor (see Table 1).

**Table 1:** The largest SPN series showing the clinical, histopathological, and post-surgical data

Year	First Author	Patient Number	Area	Ratio of malignancy	Histopathological feature	Surgical resection rate	Recurrence rate
2023	Standring O [57]	994	USA	7%	–	96.6%	–
2023	Qiaofei Liu [15]	454	China	16.5%	Vimentin, β-catenin, CD56, and alpha 1-antichymotrypsin, synaptophysin, CD10	99.3%	4.1%
2023	Jingci Chen [17]	486	China	2.4%	–	97.1%	4.3%
2022	Guangmin Wei [58]	221	China	–	–	–	3.6%
2021	Feng Yang [43]	193	China	–	β-catenin, cyclin D1, LEF1, vimentin, Ki-67 index	–	2.9%
2014	Kang [44]	341	Korea	27.9%	–	–	2.6%

### The pathological origin and mechanisms for SPN malignancy

**Fig. 2:**
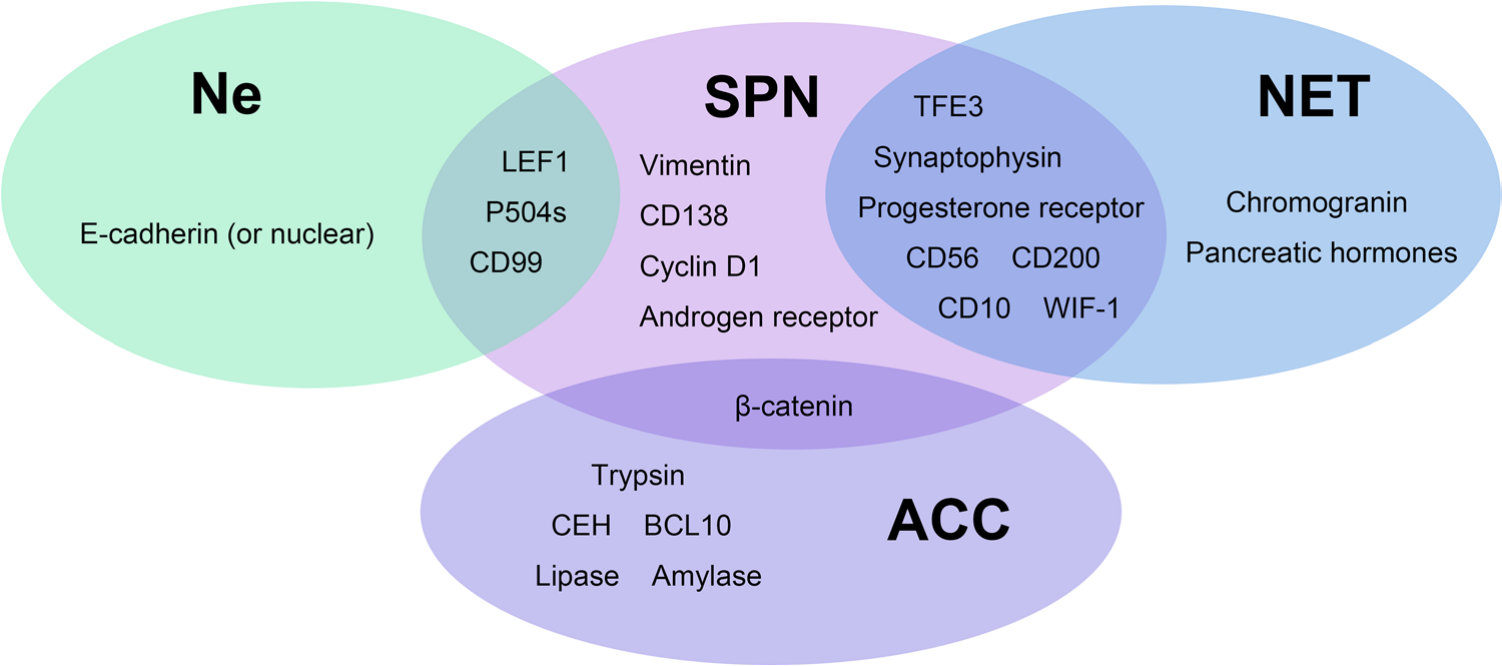
Common pancreatic tumors and their molecular markers

The pathological origin of SPN remains unclear, whereas an examination of its pathological molecular characteristics may offer valuable insights into its malignant nature. In contrast to other common epithelial-derived neoplasms in the pancreas, the pathogenic feature of SPN suggests its special origin and unique nature. Markers such as PDX1, SOX9, PTF1A, and NKX2.2, which are commonly found in pancreatic tissue, are notably absent in pediatric SPN cases [59]. Molecular markers for other pancreatic neoplasms including chromogranin A, islet hormones, amylase, GFAP, calretinin, EPCAM, and estrogen receptor α were also mostly negative in SPN (see Fig. 2). Notably, the developmental progenitors of pancreatic endocrine, ductal, and acinar cells were mostly PDX1 positive, suggesting that SPN is unlikely to be derived from pancreatic tissues [60]. In addition, mutations on p53, SMAD4, P16/CDKN2A, and KRAS, which were common in pancreatic ductal adenocarcinoma, were also rarely detected in SPN [61].

On the other hand, some unique molecular markers were frequently seen in SPN. For instance, progesterone and androgen receptors were two famous markers [54, 62]. Compared with SPNs outside of the pancreas like ovary SPNs, pancreas SPNs showed similar cytological and molecular characteristics, some of which were even highly aggressive [63–65]. Case reports showing tumor regression after menopause reinforced the relationship between SPN and the two markers [66]. Therefore, hypotheses that SPN of the pancreas was derived from the female genital bud cell lines or pluripotent cells from genital ridges attached to the pancreas were proposed [16, 67–69]. It matches the epidemiology feature of SPN by assuming it as a female-prone congenital disease. However, these theories may not be suitable to explain all SPN cases, especially for elder male patients.

It has long been discovered that nuclear and cytoplastic localized β-catenin was a hallmark of SPN observed in nearly all cases [61, 70]. The frequent mutation in exon 3 of the CTNNB1 gene was deduced to be the cause. In normal conditions, the activation of the Wnt signaling pathway releases β-catenin from the cell membrane and triggers its accumulation in the cytoplasm and nucleus, which then forms a complex with T cell factor-1/lymphoid-enhancing factor-1 (TCF/LEF1). The mutations on CTNNB1 prohibit the phosphorylation and further ubiquitination/ proteasomal degradation of β-catenin via the β-TrCP/Skp pathway during the Wnt-off period, which triggers consistent activation of downstream pathways [71]. As transcription factors, the TCF/LEF1 complex further activates downstream oncogenic genes including Myc, cyclin D1, TCF-1, CD44, GLUL, etc. [72, 73] Therefore, the Wnt/β-catenin cascade is widely involved in the regulation of cell proliferation, development, cell adhesion, and carcinogenesis [74]. Inactivated β-catenin on the membrane mediates intercellular adhesion as an E-cadherin, which explains the loose intracellular interaction in the pseudopapillary region in SPN [75, 76]. Although E-cadherin was able to be detected intracellularly, antibodies targeting extracellular fragments of E-cadherin showed negative results in SPN [54]. Interestingly, the overexpression of cyclin D1 and other related transcription factors does not promote the proliferation rate of SPN significantly, which is attributed to the simultaneous overexpression of suppressor genes p21 and p27 [77]. In summary, the mutation on the CTNNB1 gene and Wnt signaling pathway is a key feature of SPN which significantly shapes its phenotypes. It also implies the pathological origin of SPN and the malignancy potential when tumor suppressor genes are further compromised. On the other sides, certain targeted therapies for Wnt/β-catenin pathways might be beneficial for SPN patients with poor prognosis [78].

Like many other malignant tumors, the Ki-67 index was used to measure the malignancy of SPN. No standard method has been established yet for the assessment of the Ki-67 index of SPN, while most of the researched papers have employed the hot spot method. The general distribution of Ki-67 positive index ranges from 1 to 5%, most of which were less than 4% [15]. Several pieces of literature have demonstrated a strong association between an elevated Ki-67 index (generally over 4% or 5%) and unfavorable prognosis or aggressive tumor behaviors [9, 19, 58, 79, 80]. As a proliferation marker essential both in interphase and mitotic cells, the application of Ki-67 is highly recommended in the assessment of SPN [9, 81]. In addition, positive markers including AE1/AE3, CD10, Synaptophysin, Vimentin, and dotted CD99 were also applied in our institution in clinic. If these markers are used separately, there is a risk of mistaking SPN for pNET, Ne, or NCC. In a retrospective review conducted by Ohara, 30 pancreatic surgical specimens were detected for nine markers using immunohistochemical profiling. The result showed a considerable possibility of identifying SPN as pNET when synaptophysin is relied on independently [82]. The necessity to take other markers shown in Fig. 2 into account became obvious. In addition to Wnt/β-catenin signaling pathways, the expression of the Hedgehog pathway, androgen receptor signaling pathways, and several genes involved in epithelial–mesenchymal transition have been observed in SPN [53].

## The management and treatment of SPN

### The current treatment of SPN

Based on the current understanding of the biological behaviors of SPN, most guidelines hold a positive attitude toward the treatment of SPN. These guidelines assert that surgical intervention is the recommended approach for achieving comprehensive therapy of SPN, irrespective of the size of the tumor [83]. The medical community has reached no consensus on the specific method selection under disparate situations. The basic principle is choosing surgical procedures (typically radical resection) based on the difficulty of complete excision. If SPN is found to be surrounding the SMV, recommended procedures include the Whipple procedure or pancreaticoduodenectomy, otherwise distal pancreatectomy should be performed [84]. Lymphadenectomy is not commonly performed until there is evidence of lymph node infiltration on imaging, as the prevalence of such infiltration is minimal. In these cases, local lymph node dissection is typically the preferred approach. As for tumors with small diameters, complete capsules, and no metastasis, enucleation (local resection) has been increasingly used in recent years for preserving pancreatic function as much as possible, nonetheless, it increases the incidence of some complications. Wang studied 31 SPN cases managed by enucleation and summarized that enucleation reduced surgical blood loss and preserved both exocrine and endocrine functions of the pancreas. However, a greater occurrence of postoperative pancreatic leakage was observed [85]. In another study conducted by Cho, it was observed that while the overall incidence of POPF was comparable, there was a higher prevalence of severe symptoms in the enucleation group [86]. Gao concluded in a retrospective analysis which intake 194 cases that parenchyma-preserving surgery had no distinct influence on the frequency of perioperative complications or recurrence. As a result, it may be considered a more advantageous option if the overall conditions permit [87]. The advantages of local resection also include the improvement of postoperative gastrointestinal function and the reduction of mental stress. These advantages provide surgeons with the ability to tailor treatment strategies to individual patients, thereby enhancing the overall effectiveness of the surgical intervention [88].

However, the excision of malignant SPN may not always be appropriate due to its tendency to invade surrounding tissues and metastasize. For SPNs that may be surgically removed, it is recommended to do a radical resection to minimize the likelihood of recurrence. No general evaluation criteria have been proposed for the resection of SPN, while aggressive surgical resection for resectable metastatic lesions is generally accepted [52]. Radio Frequency Ablation (RFA) is also an alternative option to be considered when SPN is determined unresectable [89]. Nodal metastasis is rare in benign SPNs, whereas it is a more prevalent feature in malignant SPNs. Therefore, we recommend routine lymph node dissection in malignant SPN cases. It is crucial to promptly identify malignant SPNs before initiating therapies and provide comprehensive follow-up for such individuals. This approach hinges upon the aforementioned characteristics outlined in previous sections. Noteworthy features include: (1) integrity of tumor capsule, invasion to adjacent blood vessels and tissue, large tumor size, or metastasis under US, CT, or MRI; (2) abnormally increased glucose uptake under PET-CT; and (3) high Ki-67 index, a diffuse growth pattern, or high expression of Wnt/β-catenin pathways during pathologic examination. In certain cases, Fine Needle Aspiration Biopsy (FNAB) can be conducted as a preliminary procedure prior to surgery to get cytologic characteristics of the neoplasm, thereby aiding in the diagnostic process. However, due to the intricate nature of predicting the malignant potential of SPNs through initial basic examination, it is imperative to assemble a collaborative medical team comprising radiologists, pathologists, surgeons, and other medical professionals [90]. We recommend that SPN patients seek diagnosis and treatment from reputable large-scale medical institutions.

When the tumor exceeds resectable dimensions or when liver/peritoneal metastasis occurs, adjuvant chemotherapy drugs such as gemcitabine or 5-FU were also used tentatively to reduce the tumor volume. Given that only cases have been reported so far, its long-term efficacy remains to be further evaluated. Kang utilized the in vitro Adenosine Triphosphate-Based Chemotherapy Response Assay (ATP-CRA) to aid in the selection of chemotherapeutic agents [91]. In general, the choice of chemotherapeutic agents still depends on the individual expertise of clinicians, highlighting the pressing need for a comprehensive comparative analysis. Rarely some cases can get prolonged overall survival due to targeted chemotherapeutic interventions. Shang documented a refractory liver metastatic SPN patient who experienced significant relief under Celecoxib because of his CTNNB1 D32V mutation [92]. The utilization of genetic profiling in patients at this particular stage may offer valuable support in identifying a potential anti-cancer genetic locus and its corresponding chemotherapy regimen. This tailored approach to treatment is expected to significantly enhance patient outcomes by extending overall survival.

### Prognosis

The majority of recent studies on the prognosis of SPN did not distinguish between malignant and benign cases, while the prognosis of the former is much worse. A meta-analysis based on the survival of 59 malignant cases showed that the disease-free survival time of malignant SPN is 45 ± 6.28 months [21]. The retrospective analysis undertaken by Standring examined a cohort of 994 patients diagnosed with SPN. Its findings revealed that those with metastatic SPNs had a notably lower median overall survival (OS) in comparison to those without distant metastatic disease. Also, it has been observed that patients who exhibit clinical nodal positivity tend to experience a lower median OS compared to nodal-negative patients [57]. Numerous risk factors for malignant SPN also assist in the prediction of prognosis. Yang demonstrated a significant correlation between Ki-67 greater than 4% in postoperative pathology and poor prognosis [9]. A retrospective study conducted by Kim described the characteristics of invasive SPNs. The study identified age ≥ 40 years, PET-CT grade 3 or above (multiple or map-like hyperuptake foci with background defect), lymphatic invasion, and vascular invasion, as independent risk factors for invasive behavior [93]. In brief, malignant SPN has a prognosis that aligns with its classification as a low-grade malignant tumor by WHO. However, few patients with metastatic tumors showed good prognosis: a patient was observed to get shrinkage and disappearance of liver metastases after receiving pancreatic lesion resection in Li’s case report, which remains unclear in mechanism now [94].

## Conclusion

Being an uncommon neoplasm with inherent malignant tendencies, SPNs exhibit some characteristics in imagological, cytologic, and pathologic results when displaying malignancy. The mechanism of malignancy remains unclear, and there is a lack of consensus over the standard approach for the diagnosis of malignant SPNs is inconsistent. Typical malignant behavior encompasses infiltration, metastasis, and recurrence after R0 resection, significantly influencing tumor prognosis. The poor prognosis of malignant SPN patients has emerged as a noteworthy therapeutic challenge, necessitating the development of personalized treatment strategies that encompass more extensive surgical dissection and chemotherapy. It is recommended that the development of a comprehensive risk prediction model be undertaken to identify individuals with high-risk malignant SPNs at early stages. This approach, which has a demand for reputable large-scale medical centers, has the potential to extend the survival rates of patients diagnosed with malignancies.

## Data Availability

Data sharing is not applicable to this article as no datasets were generated or analysed during the current study.

## Abbreviations

SPN: Solid pseudopapillary neoplasm
WHO: World Health Organization
SEER: The Surveillance, Epidemiology, and End Results
RFS: Recurrence-free survival
US: Ultrasound
CEUS: Contrast-enhanced ultrasound
CT: Computed tomography
MRI: Magnetic resonance imaging
PET-CT: Positron emission tomography-computed tomography
18F-FDG: 18F-fluorodeoxyglucose
SUV: Standard uptake value
MTV: Metabolic tumor value
TLG: Total lesion glycolysis
TBR: Tumor-to-background ratio
68 Ga-FAPI: 68 Ga-fibroblast activation protein inhibitor
AJCC: American Joint Committee on Cancer
SMV: Superior mesenteric vein
POPF: Post-operative pancreatic fistula
RFA: Radio frequency ablation
5-FU: 5-Fluorouracil
ATP-CRA: Adenosine triphosphate-based chemotherapy response assay
